# Nutrition and Mental Well-Being: Exploring Connections and Holistic Approaches

**DOI:** 10.3390/jcm12227180

**Published:** 2023-11-20

**Authors:** Theodora Claudia Gheonea, Carmen-Nicoleta Oancea, Magdalena Mititelu, Elena Carmen Lupu, Corina-Bianca Ioniță-Mîndrican, Ion Rogoveanu

**Affiliations:** 1 Center for IBD Patients, Faculty of Medicine, University of Medicine and Pharmacy from Craiova, 200345 Craiova, Romania; theodora.gheonea@gmail.com (T.C.G.); ionirogoveanu@gmail.com (I.R.); 2 Department of Biochemistry, Faculty of Medicine, University of Medicine and Pharmacy from Craiova, 200345 Craiova, Romania; carmen.oancea@umfcv.ro; 3 Department of Clinical Laboratory and Food Safety, Faculty of Pharmacy, “Carol Davila” University of Medicine and Pharmacy, 020956 Bucharest, Romania; 4 Department of Mathematics and Informatics, Faculty of Pharmacy, “Ovidius” University of Constanta, 900001 Constanta, Romania; 5 Department of Toxicology, Faculty of Pharmacy, “Carol Davila” University of Medicine and Pharmacy, 020945 Bucharest, Romania; corina-bianca.ionita-mindrican@drd.umfcd.ro

**Keywords:** metabolic diseases, public health, eating habits, junk food, inflammatory bowel diseases, liver diseases

## Abstract

Quality of life, well-being, and psycho-emotional balance are closely related to the quality of the diet, the level of physical activity, the quality of rest, but also the absence of the consumption of narcotic substances and tobacco, or alcohol abuse. Based on the distribution of a questionnaire that included 30 questions, we aimed to statistically evaluate several factors that influence mental health and vices. It recorded a total of 1719 valid responses, which came from 78.3% female respondents and 21.7% male respondents. After processing the anthropometric data, it was observed that the majority of respondents are of normal weight (63.87%) and come from women in particular (36.13%). Based on the answers recorded, although over 60% of the respondents participating in the study are up to 40 years old, there is an increased tendency towards sedentarism (over 58% of the respondents declaring that they do sports very rarely or not at all), a low tendency regarding optimal consumption of vegetables and fruits, many respondents do not hydrate properly, which is why approximately 60% of respondents feel frequently tired, and over 32% are frequently nervous. The increased level of stress among the respondents and emotional eating are also generated by inadequate rest, reduced physical activity, and a diet that does not help the efficient detoxification of the body.

## 1. Introduction

In recent years, there has been an increase in research on the effects of nutrition on mental well-being, which can be a promising way to prevent or improve many mental disorders [1,2].

Recent decades have seen an exponential increase in our knowledge of the impact of the gut microbiota on human health, including brain well-being. One of the key factors involved in shaping the gut microbiota is diet, with marked effects on the diversity, abundance, and metabolic capacity of microbial organisms. Thus, eating habits influence the quality of the intestinal microbiota, and this in turn is an important pillar in the development and support of optimal mental health [3,4].

**Figure 1:**
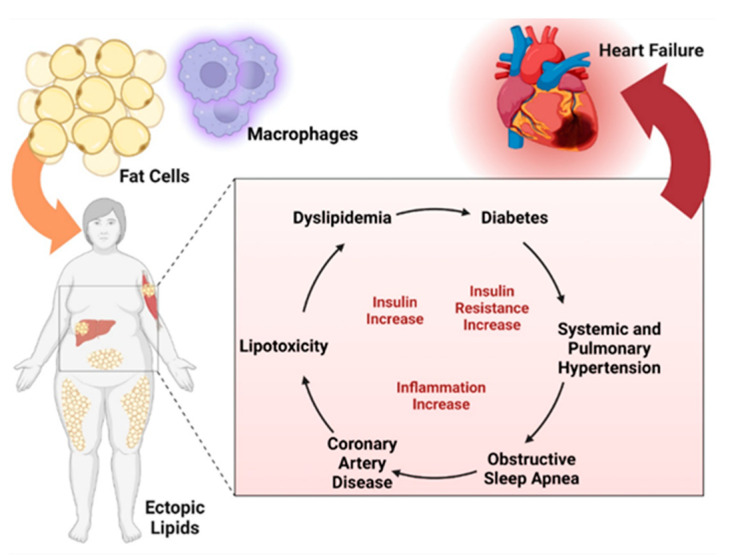
Complications of non-diabetic obesity. Created with BioRender.com (accessed on 5 October 2023).

Obesity is a serious medical condition, encountered more and more often worldwide, that requires new approaches and a recognized international consensus in the treatment of diseases that lead to morbidity. People with obesity have an increased risk of numerous complications, including hypertension, dyslipidemia, type 2 diabetes, cardiovascular disease (CVD), and various types of cancer [5,6]. These complications can be divided into several categories (Figure 1): metabolic complications (metabolic syndrome, type 2 diabetes, dyslipidemia, hyperuricemia, and gout), endocrine complications (hyperinsulinism, hypothyroidism, hypercorticism, hypogonadism), cardiovascular complications (hypertension, coronary ischemic disease, stroke, heart failure, arrhythmias and sudden death, varicose veins, deep venous thrombosis), respiratory complications (sleep apnea syndrome, asthma, dyspnea), gastrointestinal complications (non-alcoholic fatty liver, gastroesophageal reflux disease, gallstones, hernias diaphragmatic), skin complications, osteoarthritis, cancer (endometrial, breast, colon) and last but not least, depression and decreased quality of life.

**Figure 2:**
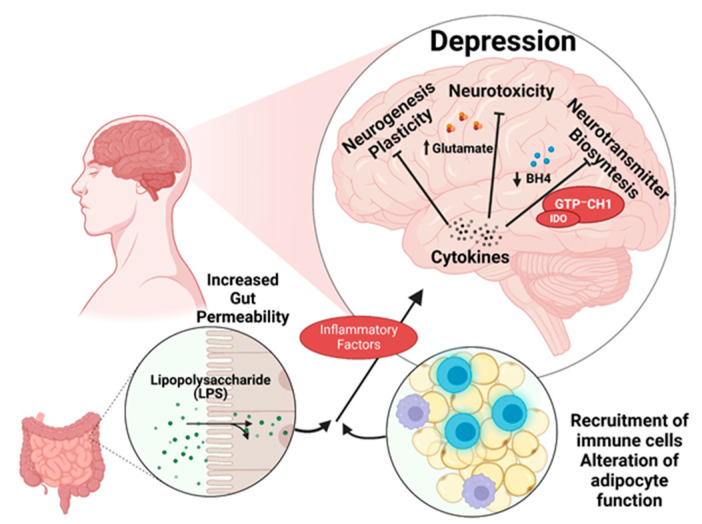
The connection between nutrition and depression. Created with BioRender.com (accessed on 5 October 2023).

A healthy diet can be beneficial in combating the pathogenesis of depression (Figure 2). As Riera-Sampol et al. have shown, both adherence to a Mediterranean-type diet and a low BMI are beneficial against depression. These positive properties of the Mediterranean diet on mental status are probably due to unsaturated fatty acids and polyphenols [7,8,9].

Other substances that are associated with a reduced risk of developing depression are polyphenols [10]. Absorption of polyphenols occurs mainly in the intestine and that process creates an interaction between polyphenols and intestinal flora. Polyphenols regulate the composition of intestinal bacteria by stimulating the growth of beneficial bacteria and inhibiting the growth of harmful bacteria. Bacteria break down polyphenols and produce polyphenol metabolites and metabolites of polyphenol-related bacteria. Short-chain fatty acids are an example of bacterial metabolites. The formation of short-chain fatty acids induces the secretion of hormones, including leptin, which has shown beneficial effects on depressive symptoms in animal experiments [11]. In addition, polyphenols may have a positive impact on neurotransmitter homeostasis. In patients suffering from depression, a reduced level of dopamine and serotonin has been observed. According to Gu et al., polyphenol resveratrol increases both dopamine and serotonin levels in a dose-dependent manner. Additionally, it increases levels of the neuroprotective molecules brain-derived neurotrophic factor (BDNF) and neuropeptide Y (NPY), which have also been shown to be decreased in depressed patients [12].

Diet is one of the key environmental factors associated with the onset and progression of inflammatory bowel diseases (IBD), which include Crohn’s disease (CD) and ulcerative colitis (UC). The evidence accumulated to date indicates the presence of an intestinal dysbiosis combined with an aberrant immune response in genetically predisposed individuals; a process likely triggered and maintained by changes in environmental factors, including diet. Because the highest prevalence of IBD is found in the Western world, it is believed that the Western diet, high in fat and sugars and low in vegetables and fruits, contributes to the development of inflammatory bowel disease, and it is also well established that the Western diet reduces the diversity of the intestinal microbiome [13,14,15].

Recent data have established the role that modern and urban diets have in inflammatory bowel diseases [16,17,18,19].

**Figure 3:**
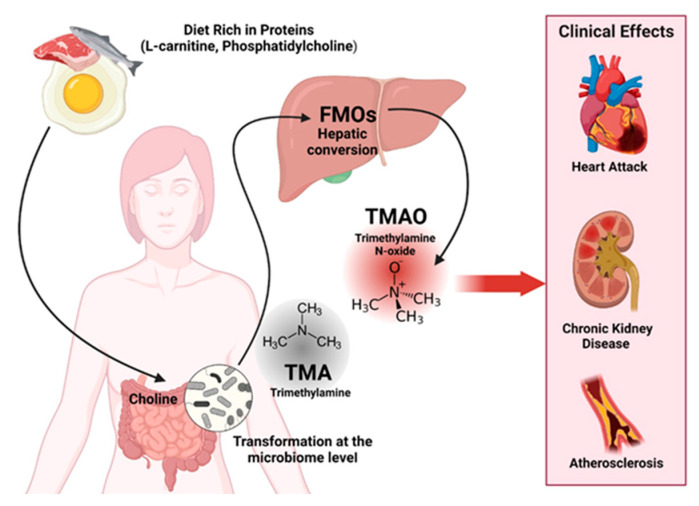
Clinical effects of diets rich in proteins. Created with BioRender.com (accessed on 5 October 2023).

A prospective study including 412 patients with ulcerative colitis in remission treated with an amino-salicylate reported that dietary intake of myristic acid, a saturated fatty acid found in nutmeg, coconut oil, and cow milk, was associated with an increased risk of relapse (odds ratio (OR) 3.01; 95% CI: 1.17–7.74). Both processed and unprocessed meats contain high levels of organic sulfur and sulfate additives, which can increase the amount of sulfate for hydrogen sulfide produced by microbes (Figure 3). The end products of protein fermentation, especially H_2_S, ammonia, and, to a lesser extent, phenols have deleterious effects on the colonic microenvironment and epithelial health [20].

In patients suffering from cirrhosis, we can often find associated depression. A suspected pathophysiological mechanism is through the accumulation of neurotoxic compounds and the interactions of some of these compounds with pro-inflammatory cytokines in the brain that diminish the ability of astrocytes to remove ammonia from the brain. Accumulation of these can interfere with the transport and activity of neurotransmitters and lead to symptoms of anxiety and depression [21]. Malnutrition is also a very common problem in people with chronic liver disease and has a negative impact on patient outcomes [22]. Cirrhotic patients show intestinal dysbiosis, have greatly reduced bacterial diversity, and overexpression of pathogens [21,23,24]. 

**Figure 4:**
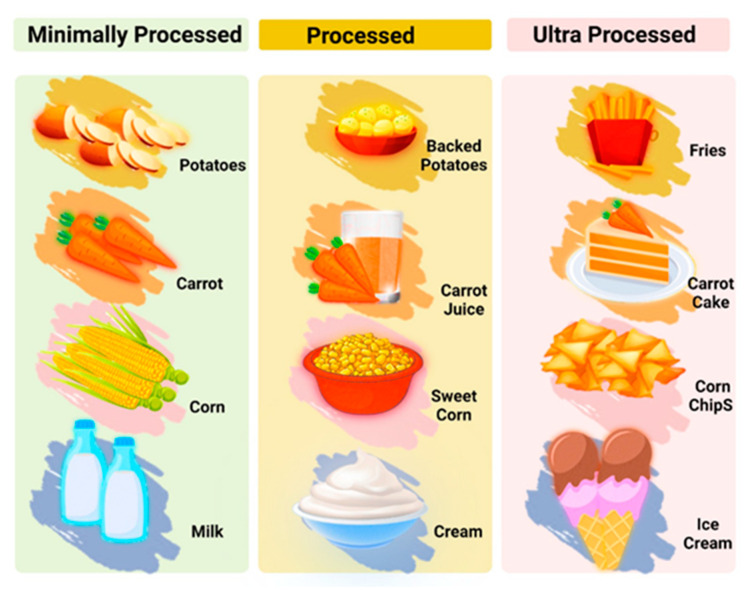
Processed food categories. Created with BioRender.com (accessed on 5 October 2023).

A large number of factors, including pleasant taste, affordability, and availability, lead people to eat highly processed foods. Individuals also consume highly processed foods in order to increase their positive emotions and reduce negative emotions [25], but in the long term the consumption of these types of foods, harmful to general health, can lead to addiction, also affecting mental well-being. The high levels of fat, salt, sugar, and artificial flavorings give ultra-processed foods intense flavors, which are not found in natural, unprocessed foods and make them highly palatable, potentially altering endogenous satiety mechanisms [26]. The potential ability of consuming highly processed foods (Figure 4) to briefly enhance positive emotions is consistent with evidence that consumption of highly processed foods trains reward and pleasure systems in a manner similar to addictive substances [27,28].

## 2. Materials and Methods

In order to analyze the influence of lifestyle and eating habits on the quality of life, a study was carried out based on the dissemination of a questionnaire with 30 questions that followed the collection of anthropometric data (height and weight), sex, age, consumption of vegetables, fruits, hydration, the consumption of junk food products, the level of physical activity and how to achieve it, the frequency of smoking, the duration of sleep, the working conditions, the factors that affect the mental state and the state of health of the respondents, the factors that affect quality of life, chronic diseases faced by the respondents, the biochemical parameters monitored (triglycerides, LDL cholesterol, blood sugar), and the frequency of health monitoring. The questions of the survey followed the assessment of the frequency and number of portions of approximately 100 g of vegetables and fruits consumed, the frequency of consumption of junk food products, the amount of water ingested daily, the frequency and number of cigarettes consumed, the duration and frequency of sports activity, the duration of sleep. The impact on the mental status and on quality of life was assessed by the existence of problems declared by the respondents in relation to the existence of frequent states of fatigue, nervousness, anxiety, depression, the presence of insomnia, emotional overeating, and lack of appetite.

The questionnaire was addressed to people aged 18 and over, participation was voluntary, with the assurance of all conditions of confidentiality of personal data and identity, each respondent agreed to participate in the study without any coercion (informed consent) and without any discrimination related to sex, religion, political beliefs, or any other nature.

To disseminate the questionnaire online, the Google Forms web platform was used through WhatsApp to the patients of family doctors [29]. The participation of the patients was carried out on a voluntary basis, without discrimination, and with complete information, with the assurance of the confidentiality of personal data. In the study, only the questionnaires of the respondents who had recent evaluations of the biochemical parameters, as well as interaction with the family doctor who monitors the state of health, were retained. The study was carried out between March and May 2023 and was approved by the Scientific Research Ethics Committee and the Scientific Council of the Carol Davila University in Bucharest (Agreement no. 188/19.01.2023) through which the protocol for maintaining the confidentiality of the data collected on the basis of the questionnaire and respecting the right to correct information of the voluntary respondents was obtained.

Data are represented as numbers and percentages in parentheses (%). A chi-square test was employed to assess the association between categorical variables. Results were significant for p value less than 0.05. Statistical analysis was performed using SPSS ver. 22.0 (IBM, Chicago, IL, USA) [30,31,32].

Anthropometric data (weight and height) were used to calculate the body mass index (BMI) using the Quetelet equation (body mass (kg)/height (m^2^)) and interpreted according to World Health Organization criteria [33].

## 3. Results

In the study based on the questionnaire, 1719 responses were recorded, of which 1346 came from female respondents and 373 from male respondents. The criterion for inclusion in the study and selection of data from the questionnaires was based on the recent evaluation of the targeted biochemical parameters as well as the recent visit to the family doctor responsible for monitoring the health status of the respondents. From the processing of the anthropometric data, it was found that 63.87% of the respondents are of normal weight, 6.13% are underweight, and 30% are overweight and obese. Regarding the age of the respondents, 61.5% of them are between 18 and 40 years old and 38.5% are over 40 years old.

For the lifestyle habits, we studied physical activity, smoking, sleep habits, and the frequency of junk food consumption, quantified by the subjects’ answers to questions (Table 1). According to the data presented in Table 1, we find a strong tendency towards sedentarism among the respondents, somewhat more pronounced in the female respondents, almost 21% of women declared that they do not do sports at all, while for men the percentage is approximately 10%. Regarding the habit of smoking, we notice that male respondents show a more pronounced tendency to smoke excessively, and the percentage of non-smokers is the highest among women (65%) compared to male respondents (56.5%). As far as the rest period, a little over half of the respondents, both women and men, have a normal sleep duration, and the rest generally get enough rest, the distribution is very similar for women and men. And in the case of the frequency of consumption of junk food products, we notice similar trends between respondents of both sexes, in general, approximately 21% of respondents consume these products very rarely. The consumption situation of vegetable products shows a low intake of these products, insufficient for respondents of both sexes, as a result, we can appreciate that there is a dietary intake deficient in fiber and antioxidants. The intake of water is not adequate either, especially for women, we find that more than 50% of them consume less than 1 l of water per day, which causes inadequate hydration of the body.

**Figure 5:**
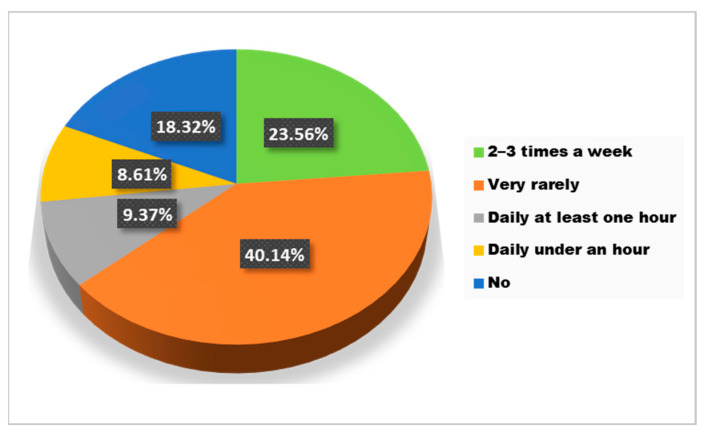
The answers to the question “Do you usually do sports/exercise?”.

According to the collected data, a statistically significant association was found between BMI with sports activities and rest periods. Regarding sports activities, 40.1% of the respondents do sports very rarely and only a percentage of 9.4% exercise at least one hour every day (Figure 5).

**Figure 6:**
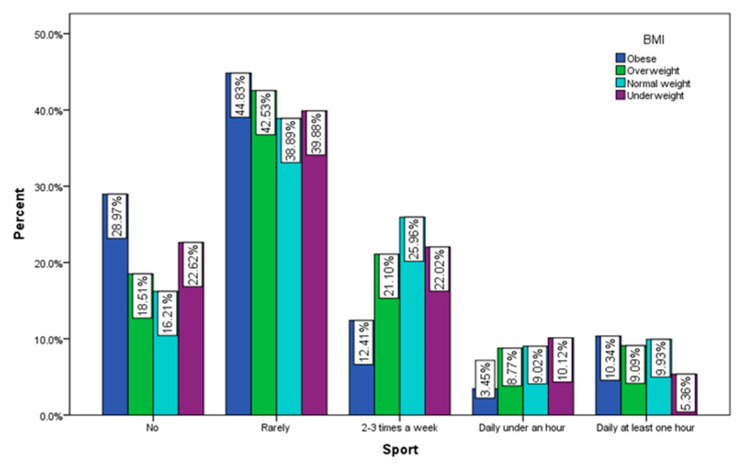
Sports by BMI (χ^2^ = 34.85, *p* < 0.001).

According to the recorded answers, 44.83% of the obese respondents rarely exercise and 28.97% do not exercise at all, 25.96% of normal weight respondents do exercise 2–3 times a week and 16.21% of them do not exercise at all (Figure 6).

**Figure 7:**
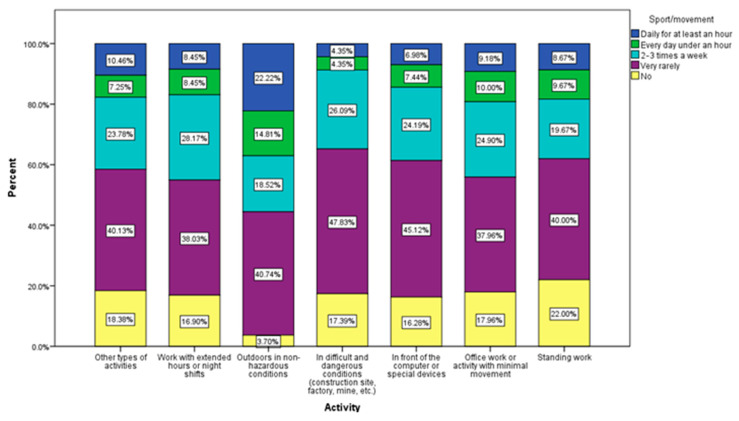
Sport/movement by profession.

We observe that those who have a job that requires field work, outside, in non-dangerous conditions, do sport/exercise every day, for at least an hour in proportion to 22.22% and only 3.7% of them do not exercise at all. On the other hand, 47.83% of those who work in difficult, dangerous conditions (construction, factories, mines, etc.), do sports very rarely. Those who work in front of the computer, those who have an office job (37.96%), or those who have a static game (40%) also do sports very rarely (45.12%) (Figure 7).

**Figure 8:**
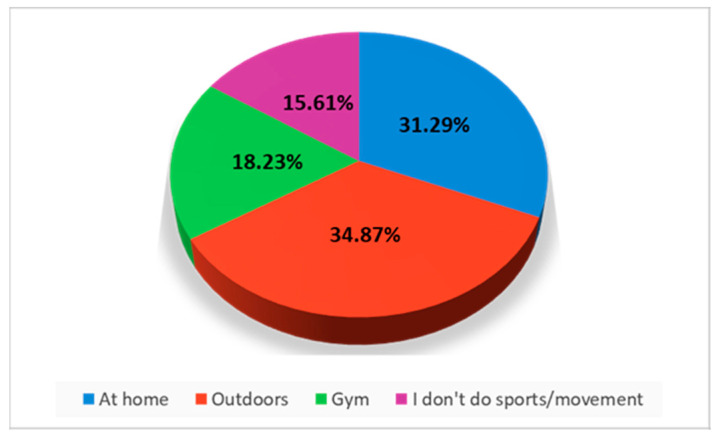
Place of sports.

The answers showed that the study participants practice sports both at home and outside, with a slight preponderance for the outdoor environment (34.87% do sports outside, and 31.29% do sports at home) and only 18.23% prefer sports halls (Figure 8).

To see if there is a relationship between sports activity, BMI, triglycerides (TG), and serum LDL cholesterol, the qualitative variables were coded as follows:

- For sports: 1—no, 2—very rarely or not at all, 3—2–3 times a month, 4—daily under 1 h, 5—daily, at least 1 h;
- For gender: 1—male, 2—female.

**Figure 9:**
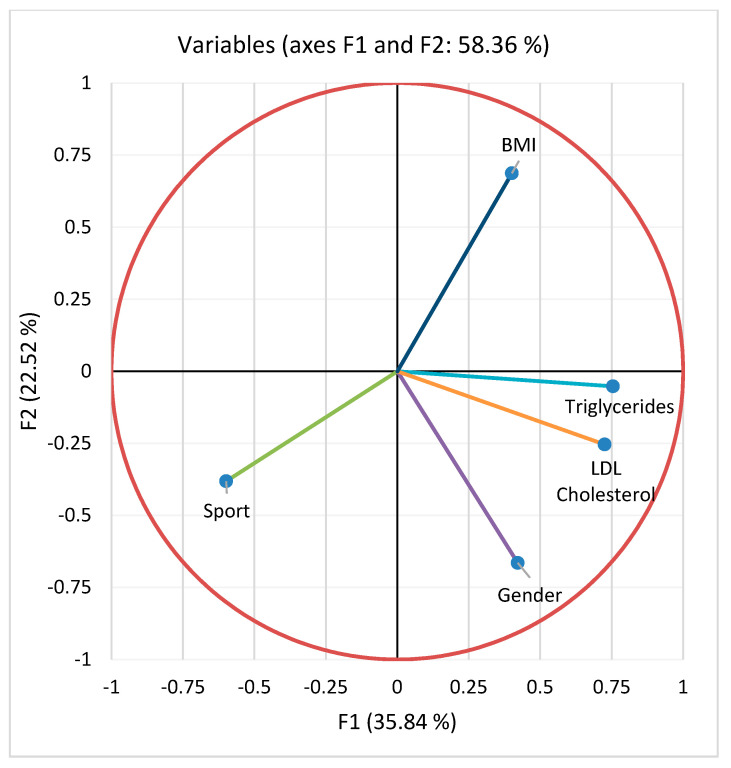
Principal component analysis (PCA) for the studied parameters: serum triglycerides, LDL cholesterol, BMI, sport, gender.

Principal component analysis was performed. A negative correlation (Table 2) can be observed between sports activity and BMI (r = −0.227, *p* = 0.018), triglyceride values (r = −0.260, *p* = 0.010), and cholesterol values (r = −0.226, *p* = 0.011) (Figure 9). Respondents who are used to doing more sports have these lower values. A positive correlation can be observed between triglycerides and LDL cholesterol (r = 0.399, *p* < 0.001).

**Figure 10:**
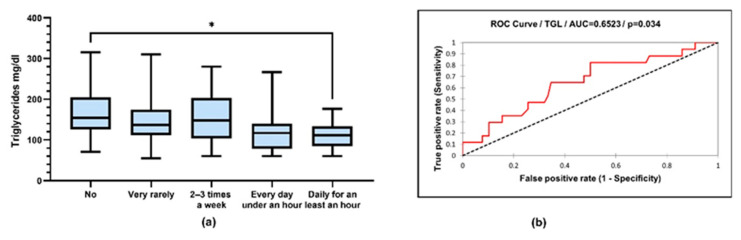
(**a**) Box-and-whiskers graph of serum concentrations of triglycerides (TG). Boxes represent 25th and 75th percentiles with mean in between, * *p* < 0.05; (**b**) ROC (receiver operating characteristic) curve of triglyceride and sports (sensitivity vs. specificity).

Triglyceride values were compared according to physical activity (TG: 170 ± 68.82 vs. 120 ± 51.31 mg/dL, *p* = 0.036; ANOVA with post hoc Tukey test) (Figure 10).

**Figure 11:**
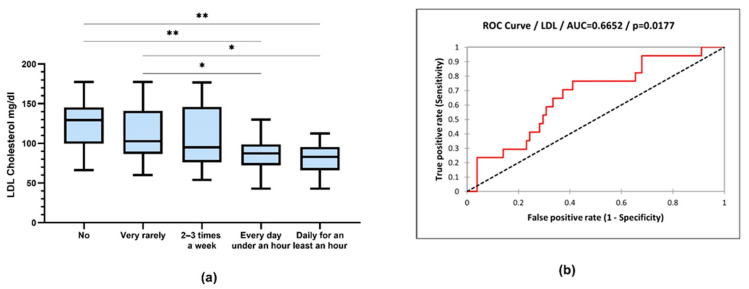
(**a**) Box-and-whiskers graph of LDL cholesterol. Boxes represent 25th and 75th percentiles with mean in between, * *p* < 0.05, ** *p* < 0.01; (**b**) ROC curve of LDL cholesterol and sports (sensitivity vs. specificity).

LDL cholesterol values were compared according to physical activity (LDL cholesterol: 125 ± 29.96 vs. 86.30 ± 22.04 mg/dL, *p* = 0.005; 125 ± 29.96 vs. 81.26 ± 20.46 mg/dL, *p* = 0.002; 112.9 ± 35.15 vs. 86.30 ± 22.04 mg/dL, *p* = 0.042; 112 ± 35.15 vs. 81.26 ± 20.46 mg/dL, *p* = 0.014; ANOVA with post hoc Tukey test) (Figure 11).

The level of sports activity is correlated with the level of serum lipids, a tendency to decrease with increasing physical activity is observed.

**Figure 12:**
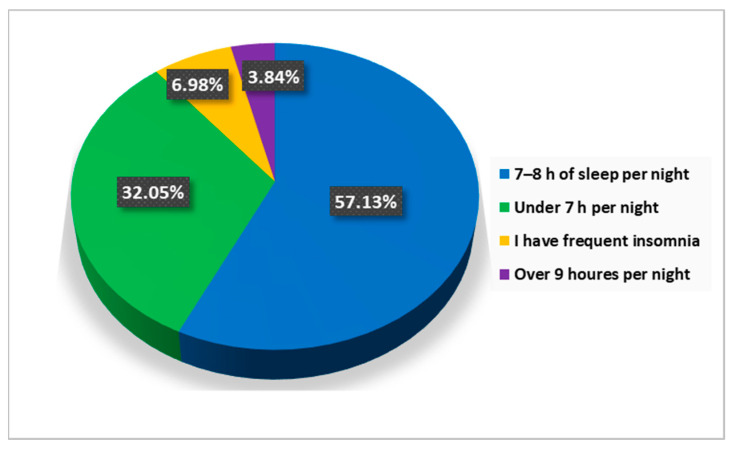
The answers to the question “How many hours a night do you usually sleep?”.

The answers to the question “How many hours a night do you usually sleep?” revealed that 57.1% of the respondents are getting 7–8 h of sleep per night, 32.1% sleep less than 7 h per night, and 7.0% have insomnia issues (Figure 12).

**Figure 13:**
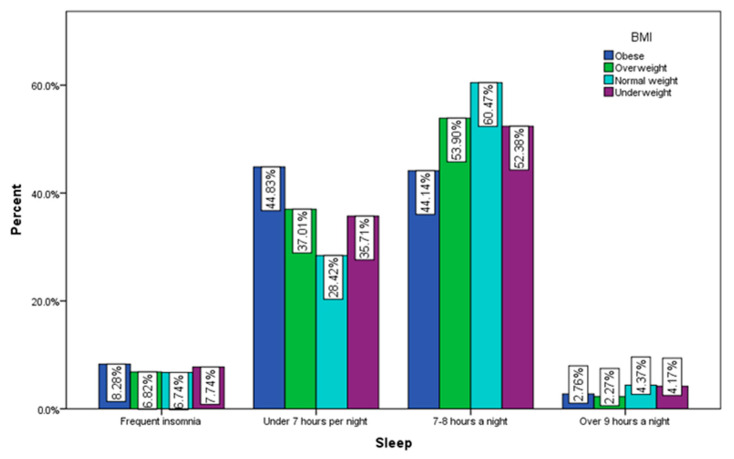
Sleeping by BMI (χ^2^ = 26.49, *p* = 0.002).

Of the respondents with a normal BMI, 60.47% declare that they usually sleep between 7–8 h a night and only 6.74% have insomnia, while only 44.14% of people with obesity sleep between 7–8 h a night and 8.28% of them face insomnia problems (Figure 13).

**Figure 14:**
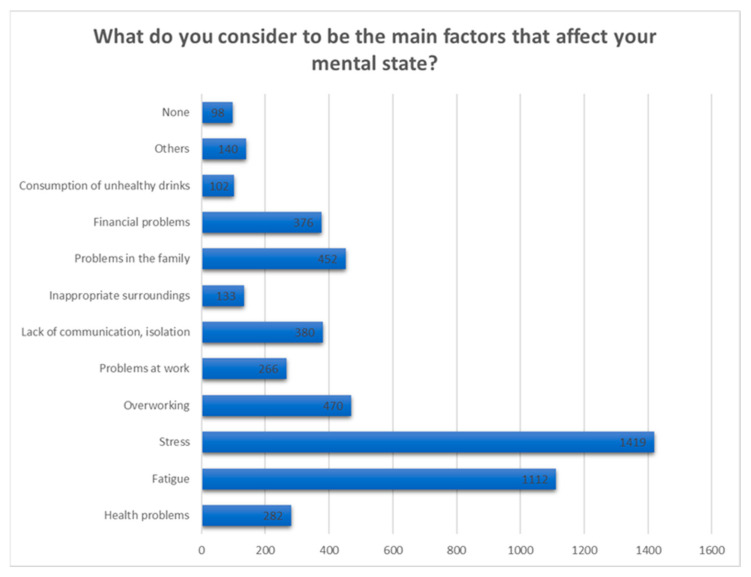
Answers to the question “What do you consider to be the main factors that affect your mental health?”.

To the question “What do you consider to be the main factors that affect your mental health?” 1419 respondents considered stress to be the most incriminated factor that disturbs their mental well-being at the moment, followed by 1112 that suffer fatigue and, respectively, by a number of 470 respondents that are mentally affected by excessive work (Figure 14).

**Figure 15:**
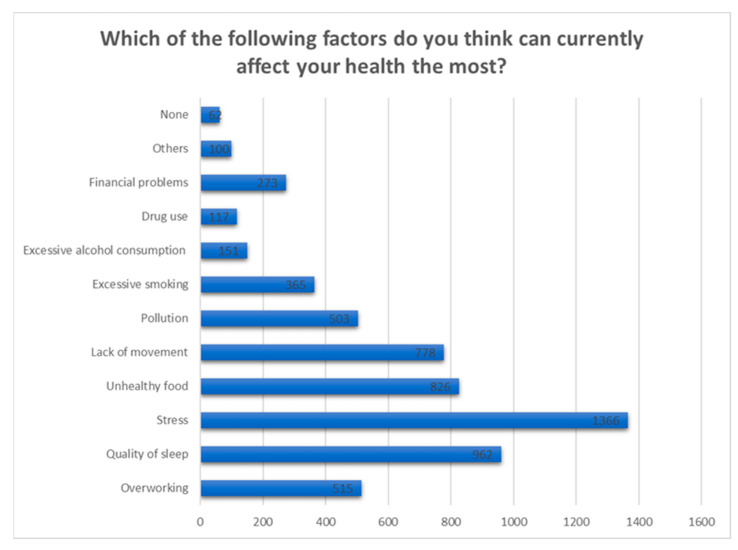
Answers to the question “Which of the following factors do you think can currently affect your health the most?”.

Also, we notice that stress is also the main reason considered by the majority of subjects to affect their general health at the moment, not only the mental one, followed by poor quality of sleep (Figure 15).

**Figure 16:**
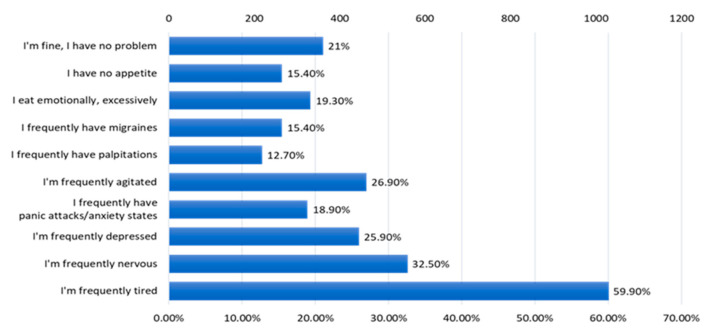
The answers to the question “What kind of problems do you encounter?”.

The majority of respondents frequently feel tired (59.90%), and 32.50% frequently experience nervousness. Also, 25.90% frequently feel depressed and 26.90% often feel agitated (Figure 16).

**Figure 17:**
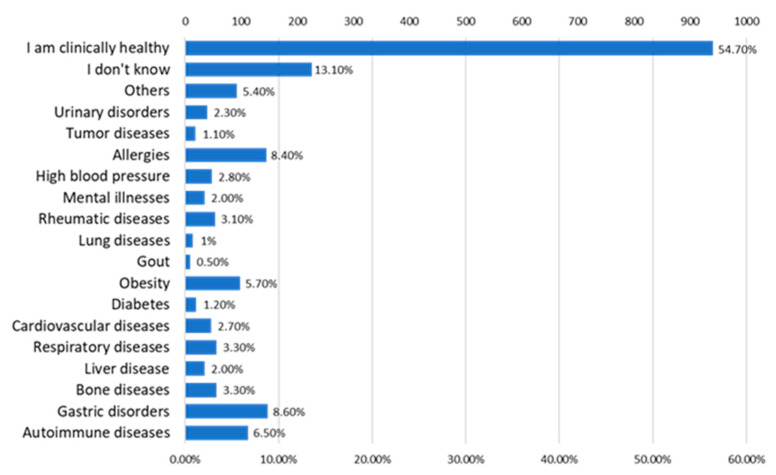
The answers to the question “What kind of chronic health problems do you have?”.

A percentage of 54.70% of the included subjects considered themselves clinically healthy, and 13.10% admitted that they did not know if they suffered from any chronic condition. Gastric diseases represented 8.60% of the respondents’ health problems, followed by allergies (8.40%), autoimmune diseases (6.50%), and obesity (5.70%) (Figure 17).

**Figure 18:**
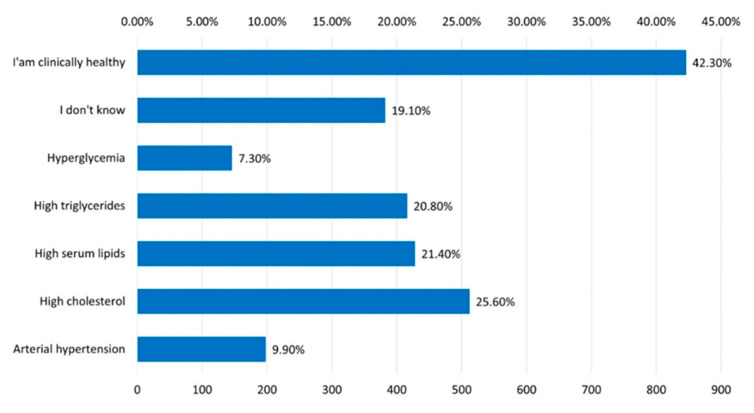
The answer to the question “What modifications of the laboratory analyzes do you know to have?”.

Regarding the para-clinical changes, 25.60% of the participants mentioned that they have high cholesterol and 21.40% are known to have increased total lipids (Figure 18).

**Figure 19:**
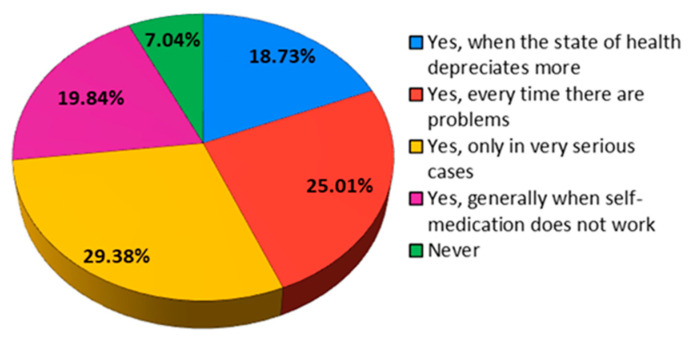
The answers to the question “Do you consult a specialist when you encounter health problems?”.

Being asked if they usually consult a specialist when they face health problems, the majority (35.78%) answered that they go to the doctor approximately once a year, and percentages of 20.54% and 22.92%, respectively, said they never go to the doctor or they go very rarely (Figure 19).

**Figure 20:**
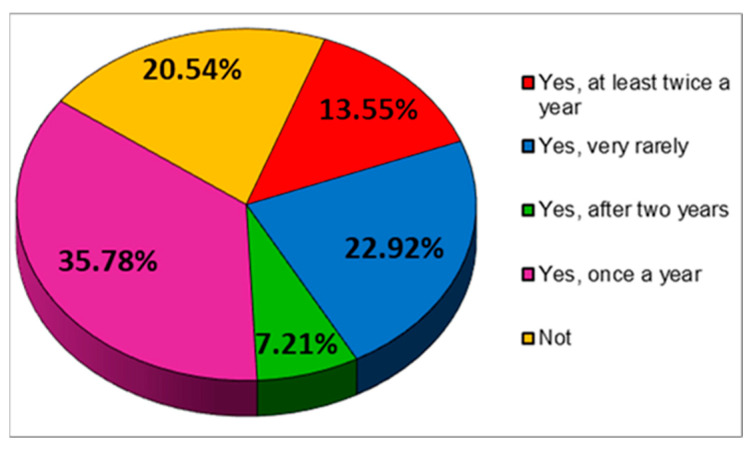
The answers to the question “Do you regularly assess your health?”.

Also, of the 1719 respondents to the questionnaire, 29.38% do checks to assess the state of health only in very serious cases of illness. A not much lower percentage, however, (25.01%) evaluates their state of health every time they encounter health problems (Figure 20).

## 4. Discussion

Although most of the respondents are young, over 60% of them are up to 40 years old, the quality of life is altered by states of fatigue (59.9% are frequently tired), frequent states of nervousness (32.5% of respondents), states of agitation (26.9% of respondents), frequent depressive states (25.9% of respondents) or anxiety states (18.9%). Only 21% of the respondents declared that they feel well, 15.4% have no appetite, and 19.3% of the respondents eat excessively on an emotional basis. It is obvious that an increased tendency towards sedentarism, an inadequate hydration of the body, a reduced intake of vitamins, minerals, antioxidants and fibers, valuable nutrients for health, an inadequate rest are the main causes of the alteration of the quality of life, and in the long term for the appearance of various ailments. It is necessary to stimulate the measures of nutritional education of the population and the promotion of physical activity. At the same time, environmental factors must be monitored, and the educated population must be involved in protecting the environment in order to protect the quality of food and health. Food safety is strongly linked to the reduction of pollutants from food sources [34,35,36].

Sedentarism, consumption of hypercaloric foods, and inadequate hydration are risk factors for the appearance of obesity, which is accompanied by an increase in BMI, an increase in serum lipids and visceral fat, and which can produce serious imbalances in the body.

Studies examining the relationships between visceral adipose tissue and cardiovascular outcomes have also confirmed that visceral adipose tissue is a huge health hazard [37]. Imaging studies have shown that visceral obesity is frequently associated with the excessive accumulation of adipose tissue at the liver tissue level, leading to the appearance of non-alcoholic fatty liver disease [38]. Other ectopic fat deposits of interest are the pericardial and epicardial adipose tissues [39]. This deposit was associated with higher BMI, traditional cardiovascular risk factors, and more atherogenic lipoprotein particles [40].

Obesity is closely related to high blood pressure, as abdominal obesity interferes with the endocrine and immune systems and carries a greater risk of insulin resistance, diabetes, high blood pressure, and cardiovascular disease. In addition, obesity is recognized as a major risk factor for hypertension in both adults and children, regardless of race, ethnicity, and gender [41].

From the processing of the collected data, a close correlation was observed between the intensity of physical activity and the serum level of lipids, with the active respondents having a much lower level of lipids. Very active people had the lowest level of serum lipids. The same trend is recorded in relation to BMI, with the most active people having a normal BMI (Figure 9). Thus, people who exercise almost every day for at least 1 h have an average serum triglyceride value of 111.9 mg/dL compared to people who do not exercise at all, whose average values were around 170.8 mg/dL (Figure 10). Regarding the level of serum LDL cholesterol, the average value of people who exercise daily for almost an hour was 81.26 mg/dL, and in the case of sedentary people, with minimal physical activity, the average value was 125.6 mg/dL (Figure 11).

Well-being is closely related to nutrition, sleep quality, physical activity, abuse of various toxic substances. From the analysis of the answers recorded, almost half of the respondents declared that they are tired or stressed (Figure 14), and among the factors blamed, the quality of food, the quality of sleep and the lack of exercise are predominantly mentioned (Figure 15).

Depression is frequently associated with obesity, metabolic syndrome, and type 2 diabetes, and there is even the question of diagnosing depression as type II metabolic syndrome. Interestingly, obesity is prospectively related to depression, and depression is predictive of the development of obesity. Thus, depression is a strong and statistically significant predictor of diet quality and body mass index, that is, higher scores in depressive symptomatology are associated with lower scores in diet quality and an increased body mass index [42,43,44,45,46].

An insufficient intake of fibers, antioxidants, vitamins, and minerals, associated with an insufficient intake of liquids, disturbs the optimal detoxification of the body, but also the health of the intestinal microbiome. These aspects stand out in the food objections of the respondents, most of them consuming small amounts of vegetable products and declaring that they hydrate inadequately (Table 1).

Disturbances of the microbiota with dietary changes or prebiotics, probiotics, or antibiotics can lead to addictive behavior or depression [47,48]. Consequently, restoring a disrupted gut microbiome could be a promising treatment strategy for depression, especially since most patients with clinically diagnosed depression additionally suffer from obesity, weight loss or gain, appetite disturbances, and constipation [34]. In rodents, the use of *Lactobacillus sp.*, *Bifidobacteria sp*., *L. helveticus*, *B. longum*, *L. rhamnosus*, *L. helveticus,* and *Lactobacillus farciminis* helped reduce symptoms of anxiety and depression [49,50,51].

In several prospective studies, a Western dietary pattern has been associated with an increased prevalence of depression [42]. Additionally, consumption of sweetened beverages, refined foods, fried foods, processed meats, refined grains, high fat intake, and baked goods have been shown to be associated with an increased risk of depression in various longitudinal studies [42].

Recent studies indicate that fruits and vegetables may be even more important to health than previously thought, and to achieve prevention of cardiovascular disease, cancer, and premature mortality, an intake even higher than the generally recommended 400 g would be needed [52]. However, the mechanism by which they influence mental health is still unclear, but there are a number of possible factors that may contribute to the positive impact. These include specific nutrients known to be related to mental health and for which fruits and vegetables are indicated as valuable dietary sources, such as complex carbohydrates and fiber, vitamin C, B vitamins, carotenoids, potassium, and polyphenols [53].

Among the most incriminated disturbing states of the psycho-emotional quality by the respondents are fatigue, nervousness, agitation, depression, anxiety, and excessive emotional eating (Figure 16). In the long term, these states can produce major imbalances in the body.

Diabetes mellitus is associated with a number of mental health consequences, including an increased predisposition to depression and anxiety, as well as decreased quality of life. Patients with poor psychological status, low social support, and low self-efficacy tend to have poorer diabetes management [54]. A 2016 meta-analysis examined psychological (stress, depression, anxiety, coping), motivational (self-efficacy), and behavioral factors (adherence to diet, physical activity, medication, self-monitoring of blood glucose, and presentation at office appointments medical) as predictors of self-care behaviors among adults with type 2 diabetes [55]. Depression contributed to significantly lower physical activity and diet adherence, while high levels of stress predicted lower medication adherence. In addition, motivational factors such as self-efficacy and coping were positively correlated with better glycemic control [54].

The mental health outcomes analyzed in these studies included quality of life, depression, anxiety, stress, and general mental health in adult patients with diabetes. The results of most studies have confirmed the positive influence of vitamin D supplementation on the mental health of people with diabetes [56]. While for health-related quality of life results were ambiguous, for depression and anxiety, vitamin D supplementation was reported to improve mental health outcomes in diabetic patients in all studies [56]. This is a very important discovery, because improvement in depression and anxiety symptoms may improve the effectiveness of antidiabetic treatment. Cardiovascular disease (CVD) is the leading cause of death in the world, and approximately three-quarters of deaths occur in low- and middle-income countries. Dietary risk factors contribute to over half of all deaths from CVD and CVD-related disability [57].

Numerous prospective cohort studies have shown that higher diet quality, assessed by various indices, is associated with a relatively lower risk of cardiovascular events or mortality [57,58,59,60,61,62,63,64]. In a 2020 systematic review and meta-analysis of prospective cohort studies, higher diet quality was associated with a 19–23% relative risk reduction in CVD incidence or CVD mortality compared to the lowest diet quality [58].

Regarding the quality of the diet, there is a tendency among the respondents of a reduced consumption of vegetables and fruits, which means a reduced intake of vitamins, mineral salts, fibers, and antioxidants from food products and a rather increased consumption tendency of type of junk food (Table 1). Nutritious foods are those that provide the nutritional elements for the health and balance of the body.

Considering that stress has a particular influence on the structural and functional aspects of the microbiome, several studies have also investigated the role of psycho-biotics in stress-related diseases. Psycho-biotics refer to probiotics or prebiotics that can manipulate the commensal gut microbiota and, when ingested in adequate amounts, can indirectly have positive psychiatric effects in psychopathology [65]. As extensively reviewed, in both experimentally induced colitis and human inflammatory bowel disease, pre and probiotics have demonstrated beneficial effects in prevention by modulating the trophic functions of the microbiota, improving the intestinal mucosal barrier, and mediating anti-inflammatory responses [66]. We can make the assumption that psycho-biotics can serve as therapeutic modulators of the gut/microbiota axis and can positively influence psychological functions in the context of inflammatory bowel diseases because the consumption of psycho-biotics seems to exert antidepressant effects, including improvements in mood and decreases in urinary free cortisol [65].

Today, food processing plays an important role in ensuring a safe, functional, and nutritious food supply. Processing can allow food to be preserved to avoid spoilage and foodborne illness. It can also increase diversity, leading to superior organoleptic properties, improved digestibility, and nutrient bioavailability. In addition, it ensures transport stability through heat treatment and allows the production of pre-prepared meals saving the time and energy required for cooking [67].

However, the ever-increasing degree of food processing is beginning to have negative effects on the health of the population, and the manipulation of tastes through additives leads to addiction, which is increasingly common, especially among children and young adults.

Approximately 75% of all mental illnesses have their onset before the age of 25. College students, in particular, are prone to depression, anxiety, and substance use disorders, and mental health problems among this population are on the rise [68]. To intervene preventively and early in their support, research in the field of nutritional psychiatry considers dietary interventions for the prevention and treatment of mental illness [69]. These interventions have the potential to contribute to improved emotional functioning and long-term health, as the adoption of a healthy diet during this developmental period can contribute substantially to the prevention of non-communicable (chronic) diseases later in life [70]. Dietary risk factors are the most important contributors to the global burden of disease, responsible for approximately 11 million deaths from non-communicable diseases (NCDs) (22% of all adult deaths) and 15% of life years with disability (DALYs) lost in 2017. The main contributors to diet-related deaths are cardiovascular disease (CVD), cancer, and type 2 diabetes [71]. Factors contributing to years of life with disability among non-fatal chronic conditions include asthma, musculoskeletal conditions, and mental health disorders [72].

Dietary risk factors involved include certain nutrients, foods, and dietary pattern exposure. Nutrient exposure includes high amounts of sodium [71,73], saturated fat, trans fat, and added sugar [73]. Dietary exposure includes small amounts of whole grains, fruits, vegetables, nuts and seeds [71], and fish [71,73], as well as large amounts of red meat, processed meat, potato chips, and sugar-sweetened beverages [73,74].

Processing can affect the nutritional (macro- and micronutrient content), physical (food structure), and chemical (presence of artificial sweeteners, newly formed additives and contaminants, glycemic index) characteristics of foods in ways that can alter their safety for health. Food processing may also influence long-term dietary behaviors, intracellular signaling pathways involved in satiety, and food reward systems [75].

In addition, the packaging used in the ultra-processed food industry doubles the health concerns as it is made from various types of synthetic chemicals and their safety has recently been questioned. Chemicals such as bisphenols and phthalates are commonly used in the production of plastic food packaging. An analog of bisphenols, bisphenol A, has been banned in many countries because of its harmful health effects, such as cardiometabolic disorders and cancer. It was replaced by another bisphenol analogue, bisphenol S, but called an “unfortunate substitution”. Bisphenol S is suspected to be absorbed orally much more than bisphenol A, and some studies have linked exposure to bisphenol S with numerous health risks, including diabetes [76].

Certain dietary patterns can also predispose to the appearance of various forms of cancer, such as the esophagus, stomach, and colorectal. Prostate cancer is a leading cause of morbidity and mortality for men [77] and although there are few studies concerning their relationship, diet and nutrition could influence prostate cancer [78]. Fast food products contain four components whose properties have been examined: salt, fat, caffeine, and sugar [79,80]. The preference for salty foods is learned from infancy between four and six months of age to develop a preference for salt based on the sodium content of breast milk, the water used to mix milk formulas, and the diet. Because high-calorie fast food products contain an increased content of salt, used for its preservative properties, a preference for salty foods is associated with higher calorie intake [81]. A study of a group of Korean teenagers showed a correlation between frequent consumption of fast food and a preference for saltier versions of traditional foods [82].

Well-being and psycho-emotional balance are also dependent on the quality of sleep. Approximately 43% of respondents do not rest properly (Figure 12), and the quality of sleep is significantly influenced by diet, caffeine consumption, smoking, and physical activity. Regarding the diet of the respondents, we find a tendency towards the consumption of junk food products at the expense of vegetable products, foods poor in valuable nutrients, and rich in saturated fats, carbohydrates, and food additives.

Reports from self-identified food addicts describe symptoms associated with sugar withdrawal as irritability, tremors, anxiety, and depression [83,84,85,86,87], symptoms also observed in opiate withdrawal. Sugar is added to foods either as sucrose, high fructose corn syrup, honey, maple syrup, or agave. Fructose appears to generate a reward response with greater toxicity than glucose [80], and sucrose administration in rodents induces behavioral changes consistent with addiction: abuse, withdrawal, craving, and cross-sensitization to other drugs of abuse.

The addictive potential of a substance is increased by increasing the dose of the addictive ingredient and increasing the rate of absorption into the bloodstream. Further research is needed to determine precisely which specific component of highly processed foods (e.g., degree of processing, combinations of macronutrients, and rapidly absorbed refined carbohydrates) is most strongly associated with withdrawal symptoms in humans [87].

Including the ubiquitous marketing of energy-dense, nutrient-poor foods is considered a strong environmental determinant of unhealthy diets and childhood obesity. Many companies are shifting advertising spend to digital marketing, where multiple promotional techniques are used to reach and engage young people online, especially on social media platforms. Such techniques include display and video advertising, direct interactions between consumers and the brand using corporate social media accounts, and active efforts to promote marketing by encouraging users to endorse companies’ promotional materials or create and share their own branded content [88].

The study carried out indicates a close dependence between the quality of food, physical activity, lifestyle, and well-being. The limits of the study are represented by the small number of male respondents.

## 5. Conclusions

In conclusion, health and mental well-being are particularly important elements both for healthy people and for those who suffer from certain comorbidities, and nutrition is one of the components of the lifestyle that can help us achieve this mental well-being. Stress was considered by the majority of respondents to the questionnaire to be the main factor that negatively influences their physical and mental health. Studies show clear evidence of direct relationships between nutrition, susceptibility to stress, mental health and mental function throughout life, and even self-care for certain existing comorbidities. The quality of nutrition, unhealthy habits can significantly affect the quality of life. This can be significantly influenced by the quality and quantity of the food products that make up the daily diet, along with the adopted lifestyle. In the long term, unhealthy food together with a disordered lifestyle can cause major imbalances in the body, accompanied by alteration of the rest process and the psycho-emotional state, which also affects the ability to work and integrate into social activities. We remark from the results of the cross-sectional observational study based on the questionnaire that the lack of movement, a diet rich in ultra-processed, hypercaloric and nutrient-poor foods, as well as an inadequate hydration of the body can affect the quality and duration of sleep and cause a state of restlessness. Chronic fatigue and even psycho-emotional damage through the appearance of nervousness or depression.

## Figures and Tables

**Table 1:** Lifestyle habits and frequency of junk food consumption with test of equality for column proportions (*z*-test).

Lifestyle Habits	Female (A)		Male (B)	
	n	%	n	%
Total	1346	78.3	373	21.7
**Exercise frequency**	**(χ^2^ = 71.14, *p* < 0.001)**			
Not	278 ^B^	20.65	37	9.92
Yes, very rarely	567 ^B^	42.12	123	32.98
Yes, 2–3 times a week	298	22.14	107 ^A^	28.69
Yes, every day under an hour	110	8.17	38	10.19
Yes, daily for at least an hour	93	6.91	68 ^A^	18.23
**Smoking**	**(χ^2^ = 15.64, *p* = 0.004)**			
Yes, excessive daily	233	17.31	98 ^A^	26.27
Yes, 1–2 cigarettes daily	91	6.76	23	6.17
Yes, 2–3 times a week	24	1.78	7	1.88
Yes, occasionally	123	9.14	34	9.12
Not	875 ^B^	65.01	211	56.57
**Sleep time, hours**	**(χ^2^ = 2.44, *p* = 0.485)**			
I have frequent insomnia	100	7.43	20	5.36
Under 7 h per night	433	32.17	118	31.64
Over 9 h a night	53	3.94	13	3.49
7–8 h per night	760	56.46	222	59.52
**Frequency of junk food consumption**	**(χ^2^ = 9.18, *p* = 0.057)**			
Very rarely or not at all	354 ^B^	26.30	75	20.11
2–3 times a month	330	24.52	84	22.52
2–3 times week	320	23.77	99	26.54
Once a week	273	20.28	91	24.40
Daily	69	5.13	24	6.43
**Servings of vegetables (approx. 100 g)**	**(χ^2^ = 7.55, *p* = 0.109)**			
Very rarely or not at all	238	17.68	88 ^A^	23.59
One	594	44.13	160	42.90
Two	368	27.34	86	23.06
Three	82	6.09	22	5.90
More than three	64	4.75	17	4.56
**Servings of fruit (approx. 100 g)**	**(χ^2^ = 91.19, *p* < 0.0.001)**			
Very rarely or not at all	272	20.21	95 ^A^	25.47
One	581	43.16	161	43.16
Two	316	23.48	73	19.57
Three	99	7.36	27	7.24
More than three	78	5.79	17	4.56
**Water consumption**	**(χ^2^ = 6.57, *p* < 0.160)**			
Less than 1 L	190 ^B^	14.12	22	5.90
1 l	509 ^B^	37.82	70	18.77
2 l	522	38.78	192 ^A^	51.47
3 l	101	7.50	65 ^A^	17.43
Over 3 L	24	1.78	24 ^A^	6.43

The values with different superscript letters (A, B) in a column are significantly different (*p* < 0.05).

**Table 2:** Correlation matrix (Spearman).

Variables	Sports	Gender	Triglycerides	LDL Cholesterol	BMI
Sports	**1.000**	−0.056	**−0.260**	**−0.226**	**−0.227**
Gender	−0.056	**1.000**	0.192	0.141	−0.049
Triglycerides	**−0.260**	0.192	**1.000**	**0.399**	0.179
LDL Cholesterol	**−0.226**	**0.241**	**0.399**	**1.000**	0.092
BMI	**−0.227**	−0.049	0.179	0.092	**1.000**

Values in bold are different from 0 with a significance level alpha = 0.05.

## Data Availability

Data are contained within the article.
